# Is intravenous lidocaine protective against myocardial ischaemia and reperfusion injury after cardiac surgery?

**DOI:** 10.1016/j.amsu.2020.09.008

**Published:** 2020-09-11

**Authors:** Chengyuan Zhang, Irwin Foo

**Affiliations:** a Department of Anaesthesia, Critical Care and Pain Medicine, Royal Infirmary of Edinburgh, Edinburgh, UK; b Department of Anaesthesia, Western General Hospital, Edinburgh, UK

**Keywords:** Lidocaine, Cardiac surgery, Myocardial ischaemia

## Abstract

A best evidence topic was constructed using a described protocol. The three-part question addressed was: In patients undergoing cardiac surgery, does intravenous lidocaine exert a cardioprotective effect against postoperative myocardial ischaemia and reperfusion injury? Using the reported search, 461 papers were found, of which 5 studies represented the best evidence to answer the question. In 3 studies, lidocaine was associated with a postoperative fall in biomarkers of myocardial injury. An additional study lacked power, but the difference in biomarkers was marginally non-significant with a trend in favour of lidocaine. A final study evaluating ischaemic changes on continuous and 12 lead ECG found no benefit with lidocaine. The limited evidence suggests that lidocaine may be cardioprotective, although no study has demonstrated improvement in clinical outcomes. Furthermore, all trials were small studies with a multitude of dosing regimens in heterogenous patient populations. There is insufficient data to correlate dose with effect and not all studies measured plasma lidocaine concentration. The narrow therapeutic index and our current evidence base does not support lidocaine prophylaxis.

## 1 Introduction

A best evidence topic was constructed according to a structured framework outlined in the International Journal of Surgery [1].

## 2 Clinical scenario

A 75-year old man is referred to you for elective cardiac surgery. You know that lidocaine has been reported to protect the myocardium from ischaemia and reperfusion injury in animal models. You consider whether a perioperative lidocaine infusion will reduce the incidence of postoperative myocardial ischaemia. To answer this question, you carry out a literature search for the evidence.

## 3 Three-part question

In [cardiac surgical patients], does [intravenous lidocaine] reduce [postoperative myocardial ischaemia]?

## 4 Search strategy

(Lidocaine.mp OR Lignocaine. mp OR Lidocaine/) AND (Cardiac Surgery. mp OR Heart Surgery. mp OR Coronary Artery Bypass Graft. mp OR Aortic. mp OR Mitral. mp OR Cardiac Surgical Procedures/ OR Coronary Artery Bypass/ OR Heart Valve Prosthesis Implantation/ OR Aortic Valve/ OR Mitral Valve/)

MEDLINE to June 2020 using the OVID interface.

## 5 Search outcome

461 papers were found using the reported search. In total, 5 randomised controlled trials (RCTs) were identified that provided the best evidence to answer the question. These are summarised in Table 1.

**Table 1:** Best evidence papers.

Author, year and country. Study type (level of evidence)	Patient group	Outcomes	Key results	Additional comments
Sunamori et al. [2], 1982, Japan Single-centre prospective RCT (level 1b)	48 patients undergoing aorto-coronary saphenous vein bypass surgery alone with CPB: Lidocaine (*L*, n = 24) No infusion control (*C*, n = 24) 1 mg min^−1^ lidocaine infusion from induction of anaesthesia up to 24 h after the end of surgery	Serum CK-MB (mean ± SEM) at 18–24 h following initiation of reperfusion	*L* vs *C*: 14.2 ± 4.0 I.U. vs 39.5 ± 15.2 I.U. P < 0.05	Hypothermia 26 °C and cold crystalloid glucose-potassium cardioplegia Patient baseline characteristics not presented Serum lidocaine concentration not measured Groups were not blinded
		Perioperative new Q waves on ECG	None in either group	
King et al. [3], 1990, Canada Single-centre prospective double-blinded RCT (level 1b)	83 patients undergoing CABG alone with CPB: Lidocaine (*L*, n = 40) Saline control (*C*, n = 43) 100 mg lidocaine bolus given at end of CPB followed by 24 h infusion of 2 mg min^−1^ Primary outcome was frequency of postoperative ventricular arrhythmias	Number of ST segment changes on ECG (>1 mm ST-depression or T wave inversion) over 24 h after surgery	*L* vs *C*: 18 vs 23	Hypothermia 25–28 °C Cardioplegia solution not stated Continuous ECG monitoring and 12 lead ECG on arrival in intensive care and at 12 and 24 h CK-MB measured with postoperative ECG changes or when clinically indicated Serum lidocaine concentration not measured
		Number of patients with myocardial infarction (new Q waves and positive CK-MB) over 24 h after surgery	*L* vs *C*: 4 vs 4 No difference in preoperative myocardial infarction between groups	
Rinne et al. [5], 1998, Finland Single-centre prospective RCT (level 1b)	100 patients undergoing CABG with CPB: Lidocaine (*L*, n = 50) No infusion control (*C*, n = 50) Lidocaine bolus 1 mg kg^−1^ before cardiac cannulation followed by infusion 1.2 mg kg^−1^ h^−1^ for 20 h	Baseline CK-MB after induction of anaesthesia and at 6 p.m. on day of surgery and the following morning	Mean (SD) CK-MB (U l^−1^): Baseline *L* vs *C*: 13 (8) vs 12 (6) 6 p.m. day of surgery *L* vs *C*: 35 (11) vs 42 (15) Morning after surgery *L* vs *C*: 44 (31) vs 51 (42) P = 0.09	Hypothermia 28–32 °C and cold blood cardioplegia No patient exclusion criteria specified No intergroup difference in preoperative myocardial infarction and ejection fraction Serum lidocaine concentration measured in single patient with severe bradycardia Groups were not blinded
		Troponin T at baseline after induction of anaesthesia and on day 3 after surgery	Mean (SD) TnT (μg l^−1^): Baseline *L* vs *C*: 0.1 (0.1) vs 0.1 (0.2) Day 3 *L* vs *C*: 0.7 (1.3) vs 1.0 (1.1) P = 0.06	
		Number of patients with myocardial infarction (two of: new Q waves, CK-MB >98 U l^−1^ and TnT >2.7 μg l^−1^) 12 lead ECG at 6 p.m. on day of surgery and morning after	*L* vs *C*: 1 vs 5 P = 0.20	
Lee et al. [6], 2011, Republic of Korea Single-centre prospective double-blinded RCT (level 1b)	99 patients undergoing off-pump CABG alone: Lidocaine (*L*, n = 49) Saline control (*C*, n = 50) Lidocaine bolus 1.5 mg kg^−1^ after induction of anaesthesia followed by infusion 2 mg kg^−1^ h^−1^ discontinued at the end of surgery	Serum Troponin I (TnI) and CK-MB at 24 h after surgery	Median (inter-quartile range) TnI (ng ml^−1^) *L* vs *C*: 0.90 (0.43–1.81) vs 1.71 (0.88–3.02) P = 0.027 CK-MB (ng ml^−1^) *L* vs *C*: 6.5 (3.9–12.3) vs 9.8 (6.0–18.6) P = 0.005	Excluded patients with hepatic diseases, serum creatinine >115 μmol l^−1^ and left ventricular ejection fraction (LVEF) < 50% Excluded patients with unstable angina and raised CK-MB or TnI, or myocardial infarction within 14 days Serum lidocaine concentrations measured before harvesting graft vessel, at the end of surgery, and 120 min after
		Total TnI and CK-MB released over 72 h after surgery calculated by area under the curve (AUC)	TnI (ng ml^−1^): *L* vs *C*: 65.8 vs 112.9 P = 0.024 CK-MB (ng ml^−1^) *L* vs *C*: 395.0 vs 538.2 P = 0.030	
		Myocardial infarction (Third Universal Definition 2012)	None in either group	
Kim et al. [9], 2014, Republic of Korea Single-centre prospective RCT (level 1b)	153 patients undergoing off-pump CABG alone: Lidocaine (*L*, n = 36) Dexmedetomidine (*D*, n = 40) Both (*LD*, n = 39) No infusion control (*C*, n = 38) Lidocaine bolus 1.5 mg kg^−1^ at induction of anaesthesia and 2 mg kg^−1^ h^−1^ infusion to 24 h after surgery Dexmedetomidine 0.3 μg kg^−1^ h^−1^ adjusted between 0.3 and 0.7 μg kg^−1^ h^−1^ to maintain mean arterial blood pressure within 20% of preoperative value	Serum TnI day before surgery, immediately postoperative and on day 1 and 2 after surgery	Median (inter-quartile range) TnI (ng ml^−1^). No significant difference between groups before surgery, immediately after and on day 1 after surgery. Day 2 (P = 0.004): *L*: 0.74 (0.39–1.37) *LD*: 0.63 (0.35–1.64) *D*: 1.43 (0.54–2.02) *C*: 1.07 (0.66–2.48) *L* vs *C*: P = 0.003 *LD* vs *C*: P = 0.048	P values obtained by Kruskal-Wallis testing. Where significant, intergroup comparisons made using one-way ANOVA with Bonferroni correction Patients with a pacemaker or medicated for arrhythmia excluded No intergroup difference in baseline preoperative myocardial infarction (>4 weeks) or LVEF Serum lidocaine concentration not measured Anaesthetists not blinded to study drug Patients, surgeon and data analyst blinded
		Serum CK-MB day before surgery, immediately after surgery and on day 1 and 2	Median (inter-quartile range) CK-MB (ng ml^−1^): No significant difference between groups before and immediately after surgery. Day 1 (P = 0.001): *L*: 7.67 (5.78–11.92) *LD*: 7.18 (5.01–11.72) *D*: 13.65 (6.89–20.61) *C*: 13.19 (6.85–23.87) *L* vs *C*: P = 0.003 *LD* vs *C*: P = 0.015 Day 2 (P = 0.000): *L*: 3.01 (1.44–4.39) *LD*: 2.83 (1.57–4.09) *D*: 5.02 (3.11–7.70) *C*: 4.84 (2.63–10.68) *L* vs *C*: P = 0.001 *LD* vs *C*: P = 0.001	
		AUC for CK-MB and TnI over 48 h after surgery	No significant difference between groups for TnI CK-MB (ng ml^−1^): *L*: 352.7 *LD*: 365.4 *C*: 566.0 *L* vs *C*: P = 0.048 *LD* vs *C*: P = 0.006	

## 6 Results

Sunamori et al. [2] compared no treatment (n = 24) with lidocaine infusion (n = 24) at 1 mg min^−1^ from induction of anaesthesia to 24 h after aorto-coronary saphenous vein bypass surgery under cardiopulmonary bypass (CPB). They found that serum creatine kinase myocardial band (CK-MB) measured 18–24 h following initiation of reperfusion was significantly lower in the lidocaine treated group (P < 0.05) (Table 1). In addition, the lidocaine treated group had a higher stroke volume index (P < 0.001) and cardiac index (P < 0.01) at 24 h after surgery. However, CK-MB levels peak at 6–8 h after injury and can decline to normal levels in 24–48 h. Thus, late measurements which fall on the declining part of the CK-MB curve may have missed or underestimated reperfusion injury.

King et al. [3] showed a significant reduction in ventricular arrhythmias (33% vs 67%; P < 0.005) in patients given 100 mg lidocaine bolus after CPB followed by 2 mg min^−1^ infusion for 24 h (n = 40) compared to placebo control (n = 43). Myocardial injury was measured through 12 lead ECG on arrival to intensive care and at 12 and 24 h. CK-MB was measured in patients with ECG changes or when clinically indicated. There was no significant difference between numbers of new myocardial infarction or ST changes on ECG (Table 1). However, studies have shown that ECG changes may be limited or absent despite significant ischaemia with an estimated sensitivity of only 45% in acute myocardial infarction diagnosed by troponin and CK-MB assays [4]. Furthermore, it is not known whether these studies achieved the therapeutic range for lidocaine as dosing was not weight based and there was no measurement of serum levels.

Rinne et al. [5] compared 1 mg kg^−1^ lidocaine bolus before cardiac cannulation followed by a 20 h 1.2 mg^−1^ kg^−1^ h^−1^ infusion (n = 50) with no treatment control (n = 50) in patients undergoing CABG with CPB. There was a clear trend towards lower CK-MB (p = 0.09), Troponin T (TnT) values (p = 0.06) and myocardial infarctions (p = 0.20) in the lidocaine treated group, but these did not reach statistical significance (Table 1). The study was underpowered by 50 patients in both groups when retrospective analysis was performed using the observed TnT difference. Serum lidocaine concentration was only measured in one patient who developed severe postoperative bradycardia with the level at the lower limit of the therapeutic range.

Lee et al. [6] compared 1.5 mg kg^−1^ lidocaine bolus after induction of anaesthesia followed by 2 mg kg^−1^ h^−1^ infusion until end of surgery (n = 49) with placebo control (n = 50) in off-pump CABG. Serum Troponin I (TnI) and CK-MB were significantly lower at 24 h after surgery and cumulatively over 72 h after surgery in the lidocaine treated group. Median TnI (inter-quartile range) at 24 h was 0.90 (0.43–1.81) vs 1.71 (0.88–3.02) ng ml^−1^ (P = 0.027) and CK-MB was 6.5 (3.9–12.3) vs 9.8 (6.0–18.6) ng ml^−1^ (P = 0.005). These associations remained significant after multivariable analysis to account for differences in baseline characteristics. Total 72 h area under the curve for TnI was 65.8 vs 112.9 ng ml^−1^ (P = 0.024) and CK-MB 395.0 vs 538.2 ng ml^−1^ (P = 0.030). This represented a 41.7% and 26.6% reduction in TnI and CK-MB respectively. Mean plasma lidocaine concentrations (SD) measured in 15 patients was 1.5 (0.3) μg ml^−1^ immediately prior to graft vessel harvesting, 2.1 (0.3) μg ml^−1^ at the end of surgery, and 0.6 (0.1) μg ml^−1^ two hours after surgery.

The therapeutic range for lidocaine when used for analgesia is described as 2.5–3.5 μg ml^−1^ whereas 2–6 μg ml^−1^ is usually quoted for its anti-arrhythmic effects [7]. It is unclear what levels should be targeted for myocardial protection. Systemic lidocaine has a narrow therapeutic index with serum levels >5 μg ml^−1^ resulting in central nervous system (CNS) toxicity. Cardiovascular toxicity can also occur when levels exceed 10 μg ml^−1^. Furthermore, lidocaine may be cardiotoxic opposed to cardioprotective at higher concentrations. In vitro studies on arterial and venous grafts have demonstrated vasodilation at low concentrations and dose-dependent vasoconstriction at higher doses [8]. Serum lidocaine concentration will also fall abruptly upon initiation of CPB due to haemodilution and increased volume of distribution. This may necessitate a significant loading dose, which may risk local anaesthetic toxicity, in order to achieve therapeutic plasma levels after bypass.

Kim et al. [9] compared lidocaine (n = 36) and dexmedetomidine (n = 40) alone and in combination (n = 39), against no infusion control (n = 38) in off-pump CABG. Lidocaine bolus of 1.5 mg kg^−1^ at induction of anaesthesia was followed by a 2 mg kg^−1^ h^−1^ infusion continued for 24 h from the end of surgery. Dexmedetomidine infusion was adjusted between 0.3 and 0.7 μg kg^−1^ h^−1^ to maintain mean arterial blood pressure within 20% of the preoperative value. Serum CK-MB and TnI were measured the day before surgery, immediately after surgery and on postoperative day one and two (Table 1). Median CK-MB concentrations on both postoperative days were significantly lower for the lidocaine (P = 0.003) and combined group (P = 0.015) compared to control. Troponin I was significantly lower for the lidocaine (P = 0.003) and combined group (P = 0.048) on postoperative day two only. The area under the curve with these time points was significantly lower for the lidocaine (P = 0.048) and combined group (P = 0.006) compared to control for CK-MB but not TnI.

There is a biochemical signal across studies grounded in scientific plausibility for lidocaine cardioprotection. Lidocaine inhibits the ischaemic induced accumulation of sodium and loss of potassium in myocardial cells [10]. Animal models have demonstrated an antiapoptotic effect after myocardial ischaemia reperfusion with reduction of infarct size and post-ischaemic improvement in functional and metabolic recovery [11,12]. However, while studies have shown significant differences in cardiac enzyme levels in favour of lidocaine, there is no direct correlate with clinical outcomes. These observed differences in biomarkers of cardiac injury therefore needs to be interpreted with caution. Studies have not been powered to detect differences in clinical outcome measures such as myocardial infarction, mortality or length of hospital stay. Those that reported rates of myocardial infarction were also poorly specified with studies utilising different definitions and methods of detection.

Lidocaine has a high hepatic extraction ratio and therefore requires dose reduction in patients with liver disease and reduced cardiac output states. Metabolites can also cause toxicity in those with heart failure and accumulate in renal failure [12]. Population studies in cardiac surgery with CPB have modelled pharmacokinetics based on body weight [13]. Prolonged infusions also require dose reduction to prevent accumulation as the active metabolite monoethylglycinexylidide (MEGX) has an inhibitory effect on lidocaine clearance. These factors were not fully explored with huge variation in dosing regimens between studies. Not all studies administered an initial bolus and concerningly, some did not consider patient weight and co-morbidities in dosing calculations.

Furthermore, not all studies measured serum lidocaine concentration making it difficult to relate dose to effect. CPB also needs to be considered as a distinct category of analysis as the pharmacokinetic differences between on-pump and off-pump surgery mandates a different approach to dosing. In addition, the risk of bias with inadequate blinding in three studies in combination with small sample sizes makes results difficult to generalise. Studies also failed to evaluate the effect of lidocaine in obtunding perioperative stress and sympathetically mediated haemodynamic changes. While hypertension, tachycardia and increased myocardial oxygen demand is clearly undesirable, any substantial drop in blood pressure will compromise coronary perfusion pressure and risk ischaemic injury.

## 7 Clinical bottom line

The available evidence is suggestive that lidocaine may be cardioprotective as there is an association with lower biochemical markers of myocardial injury in the postoperative period. However, studies were missing crucial clinical outcome data which is of practical importance to patient care. All studies were small, exclusively in coronary artery bypass surgery and with significant differences between patient groups including co-morbidities, lidocaine dosing regimen and use of CPB. This substantial heterogeneity between studies makes these findings difficult to pool or generalise. Furthermore, the optimum dosing regimen has not been established and there is a concern that inappropriately high serum lidocaine levels may be cardiotoxic. Measurement of plasma lidocaine concentrations is essential and future studies need to correlate these with not only biochemical but also clinical end points. These studies also need to be pragmatically designed and powered to prioritise objective patient-relevant clinical outcomes. With these reservations, we are unable to recommend lidocaine prophylaxis for postoperative myocardial ischaemia and reperfusion injury.

## Declaration of competing interest

No conflicts of interest to declare.

## Sources of funding

No funding has been required for this work.

## Ethical approval

Research studies involving patients require ethical approval. Please state whether approval has been given, name the relevant ethics committee and the state the reference number for their judgement.

Not applicable.

## Consent

Not applicable.

## Author contribution

Please specify the contribution of each author to the paper, e.g. study concept or design, data collection, data analysis or interpretation, writing the paper, others, who have contributed in other ways should be listed as contributors.

Chengyuan Zhang: Conceptualisation; Data curation; Formal analysis; Writing – original draft; Writing – review & editing.

Irwin Foo: Data curation; Formal analysis; Supervision; Writing – review & editing

## Registration of research studies

Not applicable.

## Guarantor

Chengyuan Zhang is guarantor for this paper.
